# Cross-Domain Fire Detection Across Indoor and Outdoor Scenes

**DOI:** 10.3390/s26103008

**Published:** 2026-05-10

**Authors:** Jingxiang Li, Xuenong Gao, Mingyang Xu, Jinzhao Zhang, Zhifeng Liu, Ruikang Luo

**Affiliations:** 1 School of Chemistry and Chemical Engineering, South China University of Technology, Guangzhou 510640, China; 202111085464@mail.scut.edu.cn (J.L.); cexngao@scut.edu.cn (X.G.); 2 School of Electrical and Electronic Engineering, Nanyang Technological University, Singapore 639798, Singapore; mxu028@e.ntu.edu.sg (M.X.); jinzhao002@e.ntu.edu.sg (J.Z.); liuz0139@e.ntu.edu.sg (Z.L.)

**Keywords:** fire detection, domain adaptation, fire detection dataset

## Abstract

Vision-based fire detection is highly sensitive to domain shifts between indoor and outdoor scenes, which often degrades the generalization of supervised models trained on a single domain. To study this problem, the Fire Detection Dataset is curated from multiple public sources as a large-scale benchmark for cross-domain fire and smoke recognition. Cross-domain deployment faces two main challenges: substantial appearance variations in fire and smoke, and highly diverse negative classes that can easily trigger false alarms. To address these issues, a tailored cross-domain framework is studied by combining adversarial alignment and discrepancy-based statistical alignment to learn more domain-invariant features and mitigate negative transfer. Experimental results show that domain adaptation substantially improves target-domain generalization over weak alignment baselines. In particular, Domain-Adversarial Neural Networks (DANN) achieve 89.44% accuracy on Indoor → Outdoor and 79.10% on Outdoor → Indoor, while Multi-Kernel Maximum Mean Discrepancy (MK-MMD) attains the best fire-class F1-score of 78.04% on Outdoor → Indoor. These results highlight the value of domain alignment for improving robust fire detection across heterogeneous deployment environments.

## 1. Introduction

This section introduces the practical motivation of cross-domain fire detection and summarizes why indoor/outdoor transfer remains challenging. Early fire detection is critical for protecting lives and property in both indoor and outdoor environments. Burns remain a major global public-health burden, with the World Health Organization estimating around 180,000 deaths annually [1]. In particular, for roadside emergency scenarios like hazardous chemical transportation, leakage incidents can rapidly escalate into catastrophic fires or explosions. Therefore, vision-based systems capable of early detection are vital for providing situational awareness and effective emergency rescue. Recent fire-detection studies have likewise emphasized the importance of improving robustness and early-warning capability in complex scenes [2]. Compared with sensor-based solutions [3], vision-based fire detection can leverage existing surveillance cameras and provide fast alarms. However, robust deployment remains challenging because visual appearance varies dramatically across domains. Indoor scenes often include cluttered backgrounds, strong reflections, artificial lighting, and occlusions, while outdoor scenes exhibit large-scale backgrounds, weather changes, and different smoke/flame patterns. Such domain shift causes supervised models trained on one domain to generalize poorly to another. To mitigate this performance degradation, Unsupervised Domain Adaptation (UDA) techniques—such as Domain-Adversarial Neural Networks (DANN) and Maximum Mean Discrepancy (MMD)—have been proposed to perform cross-domain learning. By aligning feature distributions across domains, these methods can effectively reduce domain shift and improve deployment robustness in unseen target environments.

Despite these methodological advantages, cross-domain fire detection inherently faces several significant challenges that hinder practical application. First, the appearance shift is substantial: flames and smoke vary with fuel, lighting, and imaging conditions, and background clutter differs markedly between indoor and outdoor scenes. A model trained strictly on one domain learns specific features that fail to generalize well to another. Second, the negative class (no-fire) is highly diverse; outdoor scenes include vegetation, clouds, and sunlight reflections, while indoor scenes include lamps, screens, and reflective surfaces that may mimic fire-like colors. This diversity creates severe “negative transfer” risks, easily confusing the model and leading to costly false alarms during emergency monitoring. Third, collecting and annotating sufficient target-domain data for every new deployment site is often expensive or infeasible.

To address these issues without incurring massive annotation costs, it is hypothesized that explicitly aligning the feature distributions of the source and target domains can mitigate the aforementioned shifts and extract domain-invariant fire semantics. However, it is observed that while this cross-domain task is critical, there is a severe lack of dedicated benchmarks to evaluate such domain shifts. To further facilitate research in this field, a large-scale benchmark is constructed and specifically curated for cross-domain fire and smoke recognition.

Based on this benchmark, a unified framework is designed to overcome domain discrepancies. First, it is observed that macroscopic domain variations—such as lighting and background clutter—cause severe appearance shifts. To address this, adversarial-based methods are employed to learn domain-confusing representations and handle these global appearance shifts. However, relying solely on adversarial alignment is insufficient; it often fails to align the complex, multimodal distribution of the diverse negative classes, leaving the model vulnerable to false alarms. To further improve detection reliability, discrepancy-based methods are introduced to statistically align the feature distributions at a fine-grained level. To this end, a comprehensive cross-domain fire detection framework is proposed that synergizes adversarial and discrepancy-based alignments to achieve robust generalization.

The main contributions of this paper are threefold:

- A cross-domain evaluation benchmark for fire detection across indoor and outdoor scenes is established using a curated dataset assembled from multiple public sources.
- A tailored cross-domain fire detection framework is proposed, in which macroscopic appearance shifts are handled through adversarial-based learning and negative transfer is further mitigated via discrepancy-based statistical feature alignment.
- Extensive experimental evidence is provided to show that explicit domain alignment improves cross-domain fire detection on the curated benchmark, with DANN achieving the strongest target-domain accuracy and MK-MMD yielding competitive fire-class F1 performance.

**Paper Organization.** The rest of this paper is organized as follows. Section 2 reviews related work in vision-based fire detection and unsupervised domain adaptation. Section 3 details the proposed methodology, including the problem formulation and the specific domain alignment objectives. Section 4 introduces the curated dataset, and Section 5 describes the experimental setup and training mechanisms. Section 6 reports the quantitative results, analyzes training stability, and discusses the practical limitations. Finally, Section 7 concludes the paper.

## 2. Related Work

This section briefly reviews recent progress in vision-based fire detection and unsupervised domain adaptation, with emphasis on studies most relevant to cross-domain robustness.

### 2.1. Vision-Based Fire Detection

This subsection summarizes representative fire/smoke detection methods and highlights recent studies that motivate more robust cross-scene generalization. Traditional fire detection methods rely on hand-crafted color and motion cues, while modern approaches adopt deep convolutional neural networks (CNNs) for robust representation learning. Despite strong in-domain accuracy, CNN-based fire detectors can be sensitive to dataset bias and domain shift, especially when transferring between indoor and outdoor scenes.

Early vision-based fire detection systems typically exploit color, flicker, motion, and texture heuristics, which can work in constrained environments but often suffer from false alarms under challenging lighting or background conditions. Recent deep learning approaches use CNNs to learn discriminative representations of flame and smoke directly from images or video. Representative works include CNN-based fire/smoke recognition in surveillance videos [4] and lightweight models designed for real-time deployment, such as FireNet [5]. Efficient deep CNN-based fire detection and localization in video surveillance has also been studied [6,7]. More recent studies further explore attention mechanisms, transformer architectures, and lightweight deployment-oriented designs, such as Hybrid CBAM-EfficientNetV2 for tiny-target recognition, EFNet-CSM, and MobileNetV2-based edge detection [2,8,9]. While these methods improve accuracy and efficiency, their performance may still degrade when the test distribution differs from the training data (e.g., indoor vs. outdoor scenes), motivating domain adaptation or generalization techniques.

A comprehensive review of video-based fire detection (VFD) can be found in [10], which summarizes traditional cues (color, motion, flicker) and discusses challenges such as false alarms and environmental variability. Classic real-time flame detection methods based on computer vision cues are also widely studied, e.g., [11]. Recent surveys provide a more up-to-date picture of the field, especially regarding dataset diversity, deployment constraints, and the remaining gap in generalization across realistic scenarios [12,13]. These lines of work highlight that robustness across scenes and imaging conditions remains a key bottleneck, especially when training and deployment domains differ.

Beyond image classification, modern systems increasingly adopt object detectors for fire/smoke localization in videos. General-purpose detection frameworks, such as Faster R-CNN [14], SSD [15], and YOLO [16,17], provide a practical backbone for real-time alarm systems and are commonly adapted to fire/smoke scenarios. For example, Saponara et al. deploy a YOLOv2-based real-time fire/smoke detector on embedded platforms [18]. Related detector-oriented studies have also explored improved YOLO-based surveillance fire detection and UAV-based smoke monitoring in outdoor scenarios [19,20]. Lightweight edge-oriented architectures have also been proposed, such as EdgeFireSmoke for real-time fire–smoke detection under resource constraints [9,21]. Recent surveys further emphasize open challenges on dataset bias, false alarms, and generalization across scenarios [13]. These trends suggest that robustness to domain shift remains crucial even as model architectures evolve from classifiers to detectors.

Furthermore, while these general-purpose or edge-oriented models achieve impressive real-time speeds, their reliability in safety-critical scenarios, such as roadside hazardous chemical leakages, remains a critical concern. The extreme cost of false negatives and false positives necessitates a paradigm shift. It is imperative to move beyond purely supervised learning toward domain-adaptive frameworks that can maintain high confidence and robustness across diverse and unpredictable deployment environments.

### 2.2. Unsupervised Domain Adaptation

This subsection reviews classical UDA methods together with recent survey efforts that clarify how adaptation strategies have evolved in the last few years. Unsupervised domain adaptation (UDA) aims to transfer knowledge from a labeled source domain to an unlabeled target domain. Comprehensive surveys summarize the taxonomy of deep UDA methods and their assumptions [22,23]. From a theoretical perspective, learning bounds under distribution shift motivate reducing the discrepancy between domains [24]. Domain-adversarial learning aligns feature distributions by training a domain discriminator with a Gradient Reversal Layer (GRL), such as Domain-Adversarial Neural Networks (DANN) [25]. Distribution discrepancy measures, such as Maximum Mean Discrepancy (MMD) [26] and Multi-Kernel Maximum Mean Discrepancy (MK-MMD) [27], directly minimize distance between source and target features. Correlation alignment methods match second-order statistics, such as Deep CORAL [28]. Information Maximization encourages confident and diverse target predictions by maximizing mutual information between inputs and predicted labels [29]. Discrepancy-based approaches use multiple classifiers to measure and reduce prediction disagreement on the target domain [30]. Additional representative UDA directions include deep domain confusion [31], adversarial discriminative adaptation [32], joint alignment of multiple layers [33], conditional adversarial alignment [34], cycle-consistent image translation for adaptation [35], and self-ensembling training schemes [36]. Recent survey work also highlights growing interest in practical settings such as source-free adaptation and deployment-constrained transfer, which further motivates studying robust adaptation under limited supervision [37].

Although these UDA techniques have shown remarkable success in standard image classification benchmarks, their application to specialized safety-critical tasks like fire and smoke detection is relatively under-explored. Unlike rigid objects, fire and smoke possess highly dynamic, semi-transparent visual features that easily blend with natural outdoor backgrounds or indoor artificial lighting. Therefore, this work not only benchmarks these individual UDA techniques in the context of fire detection but also proposes a synergized training objective. By leveraging adversarial learning for global background bias suppression and multi-kernel statistical matching for fine-grained feature alignment, the proposed framework is specifically tailored to meet the rigorous precision and recall demands of modern emergency monitoring systems.

## 3. Methodology

This section presents the model architecture, the role of each adaptation component, and the optimization procedure used for cross-domain fire detection.

### 3.1. Problem Formulation and Baseline Architecture

This subsection defines the cross-domain learning setting and describes the baseline architecture used throughout the experiments. In real-world emergency monitoring, the visual characteristics of fire events diverge significantly between the source training data and target deployment scenes. Let $X\subset R^{H\times W\times 3}$ denote the RGB image space and Y={0,1} denote the label space for no-fire and fire.A labeled source domain $D_{s}={\left\{\left(x_{i}^{s},y_{i}^{s}\right)\right\}}_{i=1}^{n_{s}}$ and an unlabeled target domain $D_{t}={\left\{x_{j}^{t}\right\}}_{j=1}^{n_{t}}$ are provided during training. The objective is to learn a classifier that generalizes well to the target test set under domain shift.

**Figure 1:**
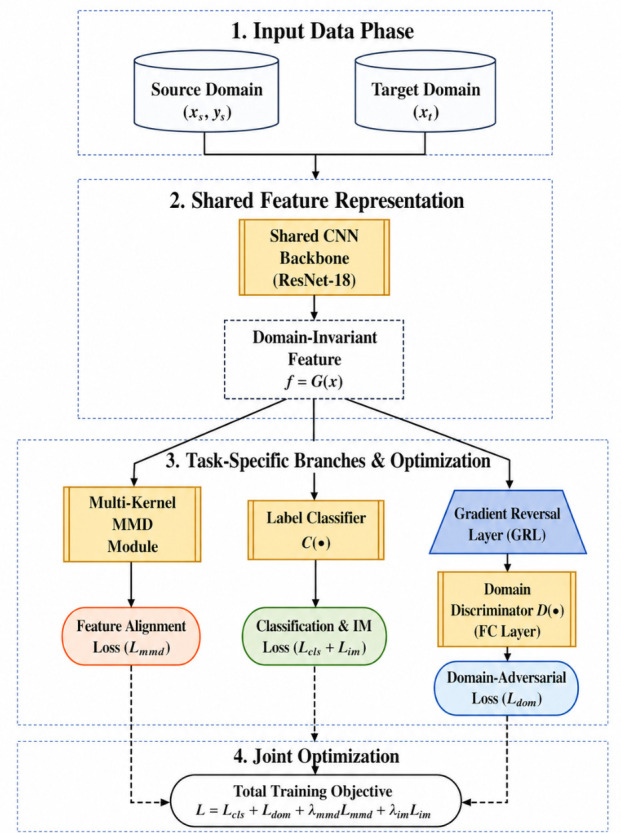
Overall framework of the proposed cross-domain fire detection method. The source and target inputs are first mapped into a shared feature representation by the ResNet-18 backbone. The shared features are then optimized through three task-specific branches: the label-classification branch, which also provides the information-maximization regularization on target predictions; the gradient-reversal-based domain discriminator for adversarial alignment; and the MK-MMD module for statistical feature alignment. The resulting losses are combined into a joint objective for end-to-end training.

A CNN-based encoder–classifier architecture is adopted. Given an input image *x*, a feature extractor G(·) (ResNet) produces a shared domain-invariant feature representation f=G(x). A ResNet backbone provides strong representation capacity via residual connections [38]. In addition, batch normalization helps stabilize optimization in deep networks [39]. A label classifier C(·) maps *f* to two-class logits for *fire* vs. *no-fire*. For domain-adversarial learning, a domain discriminator D(·) predicts whether a feature originates from the source or target domain. In the implementation, the label classifier is a linear two-class head attached to the shared feature extractor, whereas the domain discriminator is a lightweight multilayer perceptron operating on the same feature vector. As illustrated in Figure 1, the source and target inputs are first transformed into a shared feature representation, after which the classifier, GRL-based discriminator, and MK-MMD branch jointly contribute to the final optimization objective.

On the labeled source domain, standard cross-entropy is used:

(1) $$L_{cls}=E_{\left(x^{s},y^{s}\right)∼D_{s}}\left[\mathrm{CE}(C\left(G\left(x^{s}\right)\right),y^{s})\right].$$

### 3.2. Domain-Adversarial Learning for Appearance Shift

This subsection explains how adversarial alignment is used to suppress large appearance differences between indoor and outdoor scenes. A fundamental challenge in deploying fire detection models to unseen environments is the severe appearance shift. Flames, smoke, and especially background clutter vary dramatically between indoor settings and outdoor roadsides. A baseline CNN easily overfits to source-specific backgrounds, causing catastrophic failures when facing outdoor weather or vegetation.

To alleviate this domain discrepancy, it is hypothesized that the feature extractor must learn to ignore environmental distractors and focus solely on the semantic, domain-invariant properties of fire. Therefore, Domain-Adversarial Neural Networks (DANN) [25] are adopted, and a domain discriminator is trained with a Gradient Reversal Layer (GRL) to encourage domain-invariant features. Let d∈{0,1} indicate source/target. The domain loss is

(2) $$L_{dom}=E_{x∼D_{s}\cup D_{t}}\left[\mathrm{CE}(D(\mathrm{GRL}(G(x))),d)\right].$$

The standard GRL scheduling strategy is used, where the reversal coefficient increases gradually during training. Intuitively, *G* is optimized to confuse the domain discriminator, pushing source and target features closer, while preserving discriminability for the source label classifier.

### 3.3. Statistical Alignment and Regularization for Negative Transfer

This subsection introduces the additional regularizers used to reduce residual mismatch and suppress false alarms caused by ambiguous negative samples. While adversarial learning globally aligns the domains, it may fail to align the complex, multimodal distribution of the negative class (no-fire). For instance, outdoor sunset reflections or indoor red neon lights can easily trigger negative transfer, leading to false alarms. In safety-critical emergency rescue systems, such false positive rates must be strictly minimized.

To further reduce distribution mismatch, multi-kernel MMD (MK-MMD) is minimized between source and target features [26,27]:

(3) $$L_{mmd}=\mathrm{MK}\text{-}{\mathrm{MMD}}^{2}\left(\left\{f^{s}\right\},\left\{f^{t}\right\}\right),$$

where the kernel is a weighted sum of Gaussian RBF kernels with multiple bandwidths. In practice, MK-MMD measures the distance between the mean embeddings of the two feature distributions in a reproducing kernel Hilbert space (RKHS), and multi-kernel design improves robustness to scale selection. This explicit statistical alignment matches higher-order moments of the distributions, which is particularly beneficial for separating ambiguous fire-like distractors from actual fire events.

Furthermore, early detection during hazardous material leakages requires rapid and unambiguous decision-making. Information Maximization (IM) encourages confident yet diverse target predictions [29]. Let $p\left(y|x^{t}\right)=\mathrm{softmax}\left(C\left(G\left(x^{t}\right)\right)\right)$. The IM objective can be written as:

(4) $$L_{im}=E_{x^{t}}\left[H(p\left(y|x^{t}\right))\right]-H\;\left(E_{x^{t}}\left[p\left(y|x^{t}\right)\right]\right),$$

where H(·) denotes entropy. Minimizing the first term reduces per-sample predictive entropy and drives confident target predictions, while minimizing the second term encourages a non-degenerate marginal label distribution on the target batch, preventing the predictions from collapsing to a single class.

#### Overall Training Objective

The comprehensive training objective unifies the supervised classification, adversarial alignment, statistical matching, and target regularization:

(5) $$L=L_{cls}+L_{dom}+\lambda_{mmd}L_{mmd}+\lambda_{im}L_{im},$$

where $\lambda_{mmd}$ and $\lambda_{im}$ are tunable weights. In experiments, DANN, MK-MMD, discrepancy-based adaptation, and the combined DANN + MK-MMD + IM variant are benchmarked.

### 3.4. Optimization Procedure

This subsection details the training steps and clarifies how the different losses are jointly optimized. The training proceeds by sampling mini-batches from the source and target training sets in parallel. To ensure stable and unbiased gradient estimation for the domain alignment objectives, each training iteration constructs a balanced composite mini-batch comprising an equal number of source and target samples. The label classifier is trained using $L_{cls}$ on source labels, while the feature extractor is jointly optimized to (i) minimize $L_{cls}$, (ii) align features via $L_{dom}$ and $L_{mmd}$, and (iii) regularize target predictions via $L_{im}$.

The optimization pipeline in each iteration can be summarized as follows:

1. Sample one mini-batch from the labeled source domain and one mini-batch from the unlabeled target domain.
2. Feed both mini-batches into the shared feature extractor G(·) to obtain source features $f^{s}$ and target features $f^{t}$.
3. Compute the source classification loss $L_{cls}$ from the source logits and source labels.
4. Concatenate $f^{s}$ and $f^{t}$, pass them through the GRL and the domain discriminator, and compute the domain loss $L_{dom}$.
5. Compute $L_{mmd}$ between $f^{s}$ and $f^{t}$, and compute $L_{im}$ from the target predictions.
6. Form the weighted total loss and update the shared feature extractor, label classifier, and domain discriminator end-to-end using backpropagation.

Crucially, this joint optimization is achieved end-to-end via standard backpropagation. For the adversarial alignment ($L_{dom}$), the Gradient Reversal Layer (GRL) automatically inverts the sign of the gradients flowing back from the domain discriminator to the feature extractor, elegantly implementing the minimax game without requiring complex alternating update steps. Furthermore, to prevent the adaptation losses from destabilizing the early phases of representation learning, a progressive scheduling strategy is employed. Specifically, the GRL coefficient follows the standard DANN schedule

(6) $$\lambda \left(p\right)=\lambda_{\max}\left(\frac{2}{1+\exp(-10p)}-1\right),$$

where p∈[0,1] denotes the normalized training progress and $\lambda_{\max}$ is set to 1.0 in the experiments. This schedule allows the model to first establish a solid discriminative baseline from the supervised source data before aggressively penalizing domain discrepancies. Models are evaluated on the target test set across epochs, and the best checkpoint is reported for each run.

## 4. Fire Detection Dataset

This section clarifies the public data sources used to build the benchmark and summarizes the resulting domain splits and visual diversity. To rigorously evaluate the effectiveness of domain adaptation in safety-critical scenarios, there is a crucial need for a dedicated cross-domain benchmark. Since existing datasets primarily focus on single-domain recognition, a large-scale benchmark is constructed and specifically curated for cross-domain fire and smoke recognition. This section details the dataset construction, domain definitions, and statistical distributions.

### 4.1. Dataset Construction and Sources

To simulate real-world safety-critical scenarios, such as roadside hazardous chemical transportation and industrial safety monitoring, this dataset is explicitly constructed to reflect severe environmental disparities. The profound visual differences between indoor industrial settings and complex outdoor environments inherently create a substantial domain gap.

To ensure comprehensive coverage, the benchmark is curated from multiple public sources, covering diverse fire (flame/smoke) and non-fire scenes in both indoor and outdoor environments. More specifically, the fire and smoke images are assembled from individually cited public datasets, including the Kaggle datasets *Forest Fire Smoke and Non-Fire Image Dataset*, *Forest Fire Images*, and *Home Fire Dataset*, together with a Zenodo fire dataset [40,41,42,43]. The non-fire backgrounds are further supplemented using large-scale scene datasets, namely MIT Indoor Scenes and Places [44,45]. In other words, the Kaggle sources are cited as dataset references rather than citing the Kaggle platform alone, so that each source used in the benchmark remains explicitly traceable. After collection, the images are manually filtered, reorganized, and mapped into the unified binary label space (*fire*/*no-fire*) and the two deployment domains (*Indoor*/*Outdoor*). The inclusion of these large-scale scene datasets is a deliberate design choice because it provides a rich variety of background clutter, which is essential for evaluating a model’s ability to resist background-induced false alarms. To support reproducibility, the training code together with the curated source mapping and benchmark split definitions will be publicly released.

### 4.2. Domain Splits and Statistics

The dataset is organized into two domains: Indoor and Outdoor. Each domain contains two classes (fire, no-fire) and is split into train and test subsets following a standard folder structure: <domain>/(train|test)/(fire|nofire)/.

In total, the constructed benchmark comprises a massive collection of 123,696 images. The data distribution is carefully balanced to reflect realistic deployment scales:

- Indoor Domain: Consists of 59,157 images, including 40,124 fire images and 19,033 no-fire images. For the training phase, 47,323 images are allocated to the train split, leaving 11,834 images for testing.
- Outdoor Domain: Consists of 64,539 images, including 24,489 fire images and 40,050 no-fire images. The train split contains 51,481 images, while the test split contains 13,058 images.

Table 1 reports the image counts used in the experiments.

In addition, Table 2 reports the train/test split statistics used by the local experimental setup.

### 4.3. Visual Diversity and Challenges

**Figure 2:**
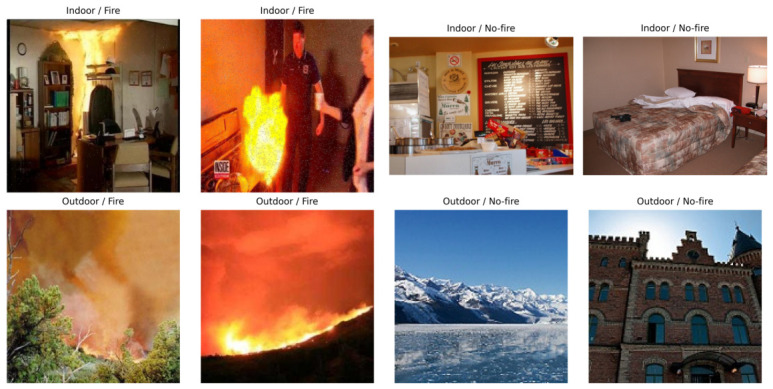
Representative samples from the curated dataset. (**Top**): indoor fire and Indoor no-fire. (**Bottom**): Outdoor fire and outdoor no-fire.

Figure 2 shows representative examples from the four domain/class combinations. The visual diversity of both the positive class (fire) and especially the negative class (no-fire) illustrates why cross-domain generalization is non-trivial.

For the positive class, flames and smoke vary drastically depending on the fuel source, lighting conditions, and the surrounding environment. For instance, indoor fires may be confined and heavily influenced by artificial lighting, whereas outdoor fires often involve massive smoke plumes and natural weather variations. More importantly, the negative class (no-fire) introduces severe “negative transfer” risks. Outdoor scenes include vegetation, clouds, and bright sunlight reflections, while indoor scenes are filled with lamps, electronic screens, and reflective surfaces that easily mimic fire-like colors. By establishing this challenging dataset, algorithms are enabled to be rigorously evaluated on their ability to maintain situational awareness without being distracted by these domain-specific backgrounds.

## 5. Experiments

This section describes the transfer directions, preprocessing protocol, optimization settings, and baseline objectives used in the empirical evaluation. Based on the newly constructed benchmark, extensive experiments are conducted to evaluate cross-domain transferability.

To comprehensively assess deployment robustness, two transfer directions are evaluated:

- Indoor → Outdoor: Trained on labeled indoor images (source) and adapted using unlabeled outdoor images (target train). Evaluated on outdoor tests.
- Outdoor → Indoor: Trained on labeled outdoor images (source) and adapted using unlabeled indoor images (target train). Evaluated on indoor tests.

### 5.1. Data Preprocessing and Evaluation Metrics

This subsection summarizes the common preprocessing pipeline and evaluation criteria shared by all methods. To build a two-domain benchmark, images are manually organized into indoor/outdoor domains and the label space is unified to two classes (*fire* and *no-fire*). All methods use a unified input size of 224×224 to match the ResNet-based backbone and ensure fair comparison across baselines. Light data augmentation (random resized crop, horizontal flip, and color jitter) is applied to improve robustness to illumination and color variations that commonly occur across domains. All inputs are normalized with ImageNet statistics to match the pretrained backbone [46].

Target-domain **accuracy** (Acc) and class-wise **precision**, **recall**, and **F1-score** are reported for the *fire* class.

Accuracy is defined as Acc=(TP+TN)/(TP+TN+FP+FN). Precision and recall for the *fire* class are defined as P=TP/(TP+FP) and R=TP/(TP+FN), and the F1-score is F1=2PR/(P+R). The *fire* class is emphasized because it is the safety-critical positive class in alarm systems.

In practical emergency response systems, missing a fire event (false negative) can lead to catastrophic consequences like delayed evacuation, while frequent false alarms (false positives) deplete rescue resources. Therefore, the F1-score provides a comprehensive and balanced evaluation of the model’s reliability and practical deployment value under domain shift.

### 5.2. Training Mechanism and Implementation Details

This subsection gives the concrete implementation details of the optimization framework and explains how the main hyperparameters are chosen. The core training mechanism of the proposed framework is designed to jointly optimize discriminative fire recognition and cross-domain feature alignment in an end-to-end manner. During each training iteration, the model receives a composite mini-batch comprising labeled source images and unlabeled target images. The optimization process is driven by two competing forces: the supervised classification loss guides the feature extractor to learn semantic representations of flames and smoke, while the domain adaptation objectives act as regularizers. These regularizers effectively force the network to discard domain-specific visual biases (such as indoor walls or outdoor vegetation). Through this dynamic training mechanism, both domains are progressively mapped into a shared, domain-invariant latent space, ensuring robust situational awareness upon deployment.

ResNet-18 [38] is used as the backbone feature extractor (initialized from ImageNet pretraining [46]). For training, images are processed with random resized crop, random horizontal flip, and mild color jitter, followed by normalization with ImageNet mean and standard deviation. For evaluation, a deterministic resize-plus-center-crop pipeline is used before normalization, still producing a final 224×224 input. This clarifies the repeated mention of image resizing: the same target input size is maintained in both training and evaluation, but the augmentation protocol differs between the two stages.

Optimization uses AdamW [47] (a decoupled-weight-decay variant of Adam [48]) with a learning of rate $5\times 10^{-5}$, weight decay $10^{-4}$, batch size 32, and 50 training epochs, together with a cosine learning-rate schedule [49]. These settings are selected to keep fine-tuning stable on the shared ResNet-18 backbone while allowing the adaptation losses to act as regularizers rather than dominate the supervised objective. The batch size of 32 provides balanced source/target mini-batches without causing unstable optimization under GPU memory constraints, and the 50-epoch budget is sufficient to observe the convergence behavior of different adaptation objectives. For the domain discriminator, a two-layer MLP with hidden dimension 256 and dropout [50] of 0.2 is used. This lightweight discriminator is intentionally chosen to provide domain supervision without excessively increasing model complexity. For the combined objective, $\lambda_{mmd}=0.1$ and $\lambda_{im}=0.1$ are set, and RBF kernel bandwidths {1,2,4,8,16} are used for MK-MMD. These weights are selected empirically to keep the discrepancy- and entropy-based regularizers on a comparable scale to the source classification term, thereby improving alignment while avoiding noticeable degradation of source discrimination. The GRL coefficient follows the progressive schedule defined in Section 3, with $\lambda_{\max}=1.0$.

To provide a clear and comprehensive overview of the experimental setup, all key hyperparameter configurations utilized in our framework are summarized in Table 3.

Among these configurations, the adaptation trade-off weights $\lambda_{mmd}$ and $\lambda_{im}$ are particularly critical, as they directly govern the balance between domain-invariant feature alignment and source-task discriminability. Rather than selecting these values arbitrarily, their optimal values were determined through a small-scale parameter sensitivity analysis on the target validation set. Table 4 illustrates this analysis conducted on the Indoor → Outdoor transfer direction across a selected range of representative values {0.01,0.1,0.5,1.0}.

Based on this sensitivity analysis, we set both $\lambda_{mmd}$ and $\lambda_{im}$ to 0.1, as this combination achieved the optimal balance and yielded the highest Accuracy (88.53%) and F1-score (87.79%). It can be observed that either under-regularization (weights set to 0.01) or over-regularization (weights set to 0.5 or 1.0) leads to performance degradation. Specifically, excessive adaptation penalties can dominate the total loss, forcing the feature extractor to over-align the domains at the expense of losing essential fire-specific semantics. These parameters remained fixed for all subsequent experiments to ensure a fair comparison across different transfer directions.

### 5.3. Baselines Adaptation Objectives

This subsection clarifies the adaptation objectives compared in the experiments. To comprehensively benchmark the effectiveness of cross-domain fire detection, the following adaptation objectives are carefully selected to represent mainstream philosophies in unsupervised domain adaptation:

- **Maximum Mean Discrepancy (MMD)**: feature alignment using (single-kernel) maximum mean discrepancy.
- **Multi-Kernel Maximum Mean Discrepancy (MK-MMD) Baseline**: feature alignment using multi-kernel MMD.
- **Domain-Adversarial Neural Networks (DANN) Baseline**: domain-adversarial feature learning with a gradient reversal layer.
- **Maximum Classifier Discrepancy (MCD)**: classifier discrepancy minimization to reduce disagreement on target predictions.
- **DANN + MK-MMD + Information Maximization (DANN + MK-MMD + IM)**: a combined objective that integrates adversarial alignment, MK-MMD alignment, and information maximization.

## 6. Results and Analysis

This section presents a comprehensive analysis of the experimental results. First, the main quantitative results are reported to compare the target-domain performance of different adaptation objectives. Then, qualitative visual examples are provided to illustrate typical success and failure cases under indoor–outdoor domain shifts. The training dynamics are further analyzed to explain the convergence behavior and best-checkpoint selection of different methods. Finally, the asymmetric difficulty between the two transfer directions is discussed, followed by a brief analysis of the limitations and practical deployment implications of the proposed framework.

### 6.1. Main Results and Methodological Effectiveness

This subsection summarizes the quantitative comparison and discusses what can be concluded from the current benchmark without overstating the evidence. Table 5 summarizes the best target-domain performance for each method.

Overall, domain adaptation improves cross-domain generalization compared with weaker alignment baselines (e.g., MMD), confirming that explicit feature alignment is beneficial in this benchmark. Among the reported methods, DANN achieves the best target-domain accuracy in both transfer directions, reaching 89.44% on Indoor → Outdoor and 79.10% on Outdoor → Indoor. MK-MMD also yields strong performance, while discrepancy-based adaptation (MCD) is less stable in this setting.

**Figure 3:**
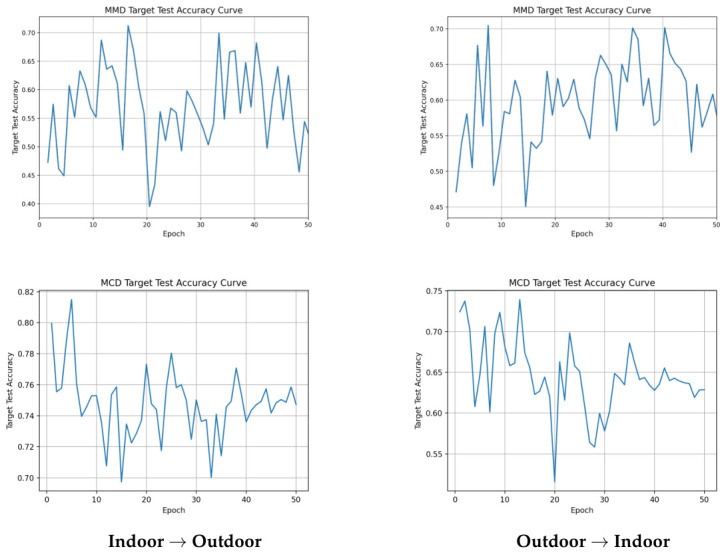
Baseline training accuracy curves (part I). (**Top**): MMD. (**Bottom**): MCD. (**Left**): Indoor → Outdoor. (**Right**): Outdoor → Indoor.

**Figure 4:**
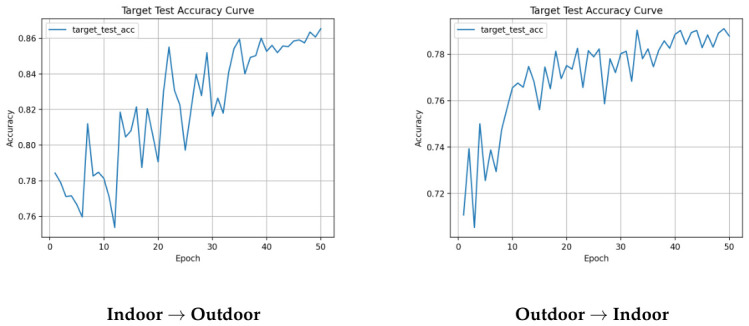
Baseline training accuracy curves (part II). (**Left**): MK-MMD on Indoor → Outdoor. (**Right**): DANN on Outdoor → Indoor.

From a class-wise perspective, DANN achieves a strong *fire* F1-score on Indoor → Outdoor (89.06%), indicating both high recall and precision for the safety-critical class. On Outdoor → Indoor, MK-MMD slightly improves the *fire* F1-score to 78.04% while matching the best accuracy of 79.10%, suggesting that kernel-based alignment can provide a better trade-off between false alarms and missed detections in this direction. In contrast, MMD yields notably lower accuracy (71.30% and 70.47%) and shows unstable learning behavior (Figure 3 and Figure 4), highlighting the difficulty of aligning complex visual domains with a weak discrepancy objective.

To understand the source of these performance gains, the progressive effectiveness of different adaptation modules is analyzed. As established in Section 1, the primary challenge in cross-domain fire detection for emergency scenarios is the severe appearance shift and background clutter. Relying solely on a simple discrepancy objective (such as single-kernel MMD) struggles because it is insufficient to bridge the complex distribution gap of fire and non-fire distractors. In contrast, introducing a more fine-grained discrepancy-based alignment via MK-MMD captures higher-order moments and improves target-domain generalization.

Furthermore, when an adversarial-based learning objective is employed, the highest performance leap is observed in terms of target-domain accuracy. This validates the initial motivation that adversarial learning helps the feature extractor suppress domain-specific background cues and retain more transferable fire semantics. The combined DANN + MK-MMD + IM objective also performs competitively on Indoor → Outdoor (88.53% Acc and 87.79% F1), indicating that confidence regularization and statistical alignment remain useful components, although the current table does not support claiming uniform superiority over all baselines in every setting.

Compared with recent fire- and smoke-detection studies reported in the literature [2,8,20], the present benchmark addresses a more difficult cross-domain transfer setting rather than standard in-domain recognition on a single dataset. Therefore, the comparison with prior work should be interpreted mainly at the level of problem setting and robustness objective: many recent methods report strong accuracy under dataset-specific evaluation, whereas Table 5 shows that substantial performance degradation still occurs once training and deployment domains differ. This observation further supports the need for adaptation-oriented evaluation in fire detection.

### 6.2. Qualitative Visual Analysis

**Figure 5:**
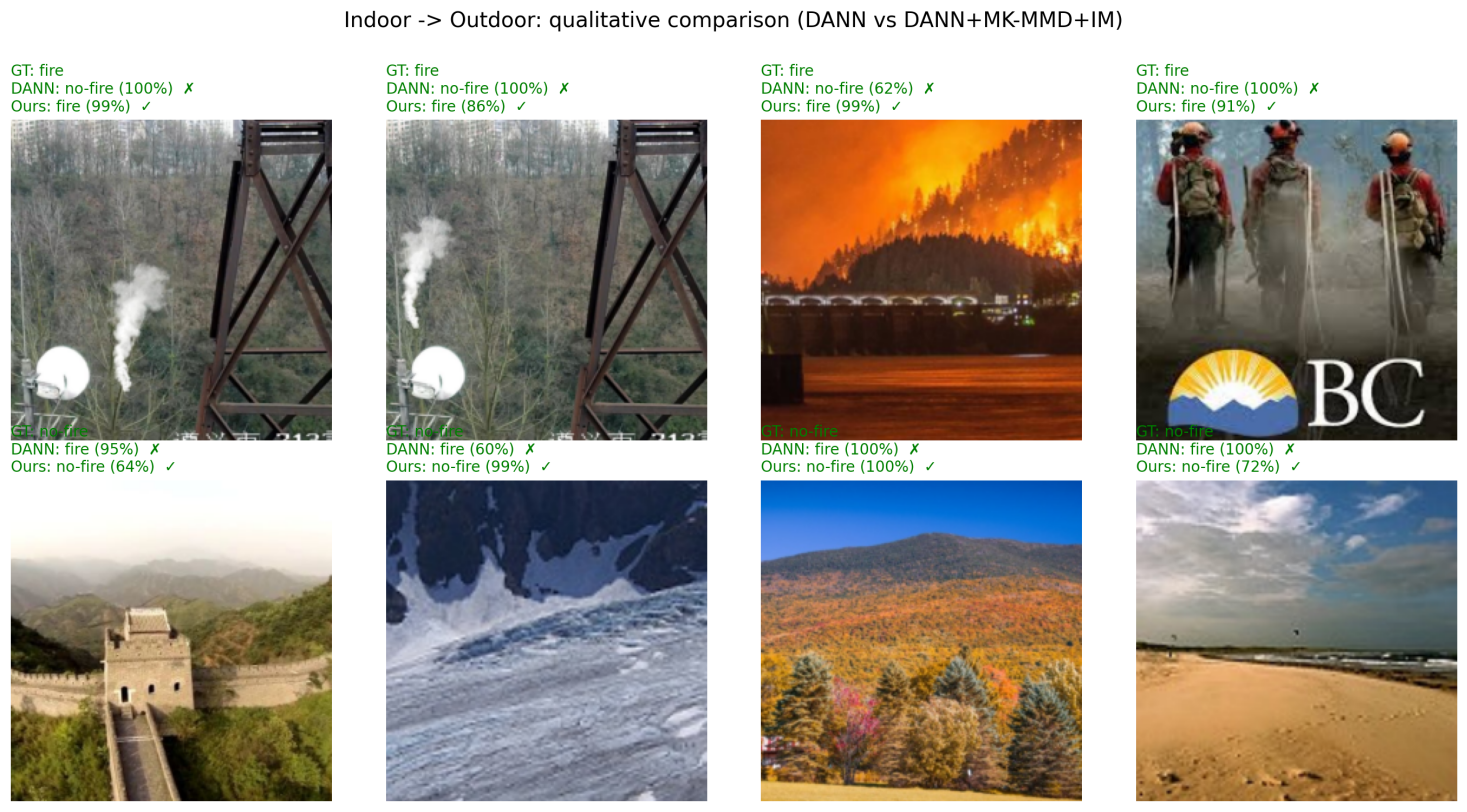
Qualitative comparison on the Indoor → Outdoor transfer direction. Each column shows a target-domain test image together with the DANN baseline prediction (taken from the last training epoch) and the DANN + MK-MMD + IM prediction (taken from its best checkpoint). (**Top**): fire ground truth; (**Bottom**): no-fire ground truth. The selected samples highlight cases where the proposed framework corrects DANN failures even after full training.

**Figure 6:**
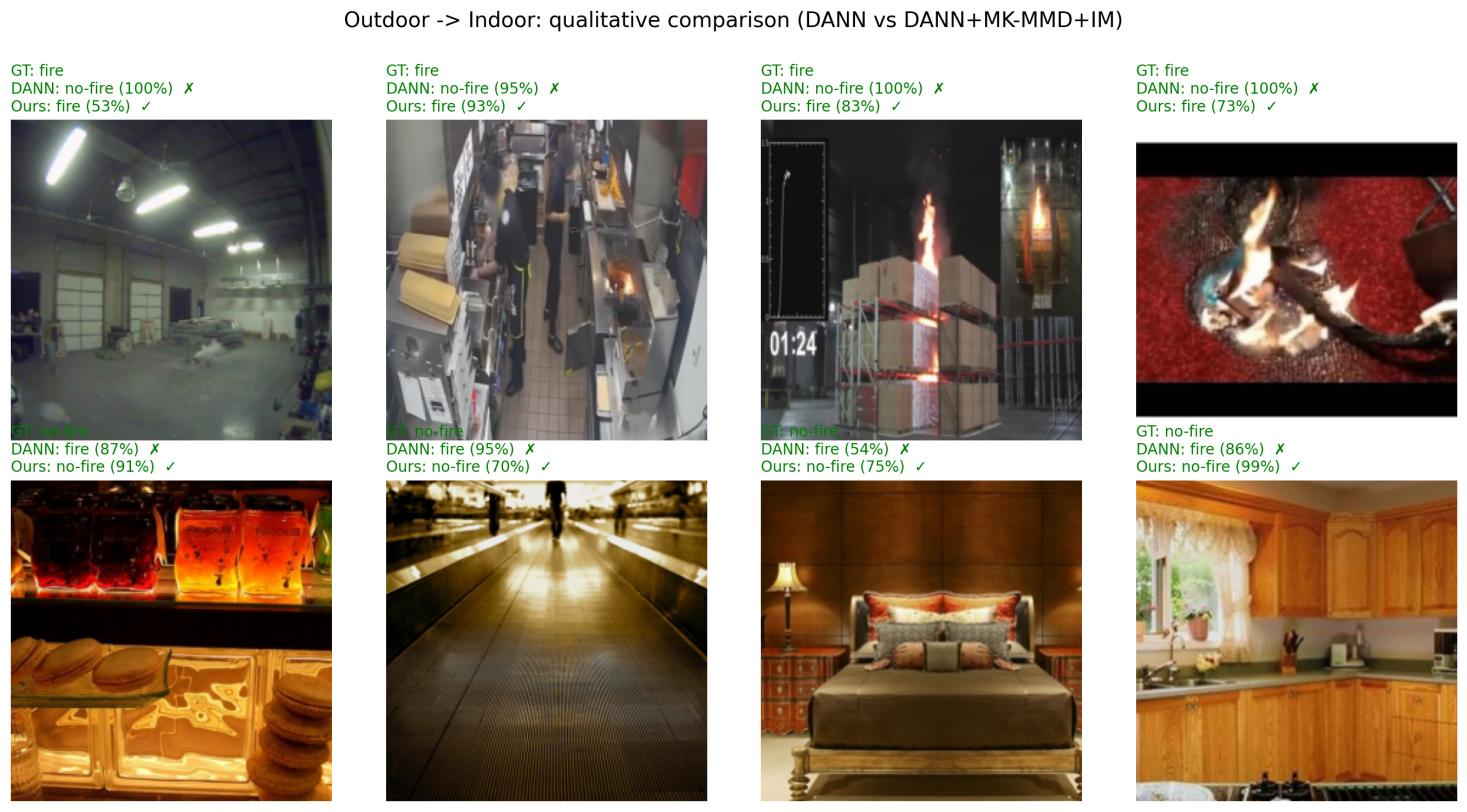
Qualitative comparison on the Outdoor → Indoor transfer direction. Each column shows a target-domain test image together with the DANN baseline prediction (taken from the last training epoch) and the DANN + MK-MMD + IM prediction (taken from its best checkpoint). (**Top**): fire ground truth; (**Bottom**): no-fire ground truth. The selected samples highlight cases where the proposed framework corrects DANN failures even after full training.

To intuitively demonstrate the effectiveness of the proposed framework and to address the limitations of conventional baseline models, we conduct a qualitative visual analysis of the detection results. Figure 5 and Figure 6 visualize representative target-domain samples from the Indoor → Outdoor and Outdoor → Indoor transfer directions, respectively. In each figure, a 2×4 grid of samples is presented, comparing the predictions of the DANN baseline against our combined DANN + MK-MMD + IM framework. The top rows represent fire scenes (testing for false negatives), while the bottom rows depict non-fire scenes with complex backgrounds (testing for false positives).

As observed in the visual results, the conventional DANN baseline struggles with two major failure modes. First, in the presence of target-domain-specific weather or lighting conditions (e.g., fine smoke in the forest or low-contrast indoor illumination as shown in the top rows), DANN fails to detect the fire signals. Second, and more critically, DANN is easily confused by hard negative distractors, falsely triggering alarms on sunset glow, mountain reflections, or indoor warm-colored lighting (bottom rows). This occurs because DANN relies solely on global adversarial alignment, which is insufficient to match the fine-grained, multimodal distribution of the highly diverse negative classes.

In contrast, our proposed framework successfully corrects these misclassifications with high confidence. The superiority of our approach lies in the integration of discrepancy-based statistical alignment (MK-MMD) and Information Maximization (IM). While adversarial learning handles the macroscopic appearance shift, MK-MMD explicitly aligns the higher-order statistical moments of the features, successfully separating ambiguous target-domain distractors from the actual fire semantics. Coupled with the IM module that enforces confident target predictions, the proposed model significantly reduces the false alarms that plague the baseline model, highlighting its robust performance in safety-critical deployment scenarios.

### 6.3. Training Dynamics and Stability Analysis

This subsection analyzes convergence behavior and explains the reported training schedule in relation to the best-checkpoint protocol. To make training variability explicit, Table 6 reports the best epoch and the corresponding metrics for each run. All methods are trained for 50 epochs using AdamW with a learning rate of $5\times 10^{-5}$, but the best checkpoint appears at different epochs across methods and transfer directions, motivating best-checkpoint reporting rather than using a fixed final epoch.

**Figure 7:**
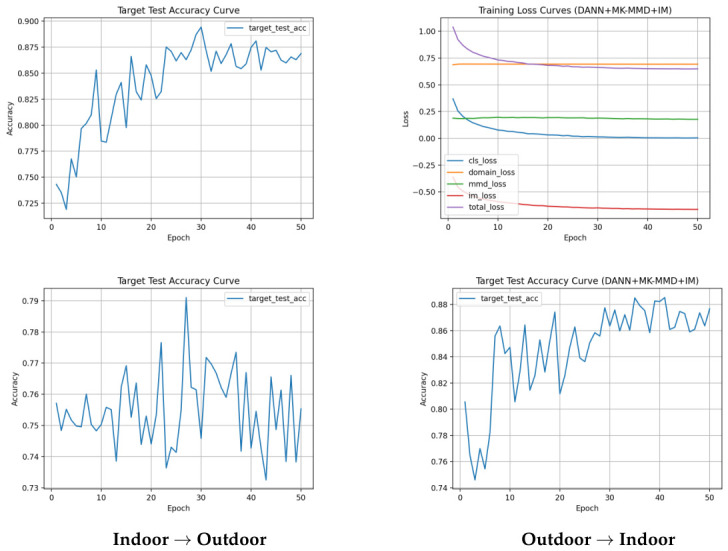
Training curves for the proposed method (DANN + MK-MMD + IM). (**Top**): loss components. (**Bottom**): target test accuracy. (**Left**): Indoor → Outdoor. (**Right**): Outdoor → Indoor.

Detailed training curves exported from the simulation runs are provided in Figure 3, Figure 4 and Figure 7 to facilitate inspection of training stability. Since target-domain performance can fluctuate across epochs, the best checkpoint per run is reported in Table 5.

The training curves illustrated in Figure 3, Figure 4, and Figure 7 further clarify the learning process. The fluctuating accuracy observed in weaker baselines (such as MCD or single MMD) reflects the model’s difficulty in finding a stable decision boundary under severe domain shift. By contrast, the stronger baselines reach their best checkpoints at different stages of training: for example, DANN peaks at epoch 30 on Indoor → Outdoor and epoch 27 on Outdoor → Indoor, MK-MMD peaks late at epochs 50 and 49, while MCD reaches its best checkpoint earlier at epochs 5 and 13 (Table 6). These observations indicate that the same 50-epoch training budget and learning-rate schedule can lead to very different convergence patterns depending on the adaptation objective. The combined DANN + MK-MMD + IM framework also exhibits comparatively smooth loss evolution on Indoor → Outdoor, suggesting that the joint objective can stabilize optimization when the source-to-target transfer is less adverse.

### 6.4. Analysis of Asymmetric Domain Shift

An asymmetric domain shift is observed: the best achievable accuracy on Indoor → Outdoor is substantially higher than that on Outdoor → Indoor (Table 5). One plausible explanation is that indoor scenes exhibit broader illumination and background variations (e.g., reflections, screens, and clutter), and the *no-fire* class indoors contains more visually confusing patterns, making Outdoor → Indoor transfer harder. This asymmetry indicates that cross-domain robustness should be evaluated in both directions rather than reporting a single transfer setting.

From the perspective of practical deployment in emergency rescue, this asymmetry is highly informative. It suggests that models trained on highly complex indoor environments (which contain rich artificial distractors) inherently learn more robust representations that transfer well to outdoor scenes. Conversely, outdoor-trained models may overfit to specific natural backgrounds. Therefore, for broad roadside and industrial applications, curating source datasets with maximum background diversity is a critical prerequisite for reliable deployment.

### 6.5. Limitations and Practical Deployment Discussion

This work focuses on image-level binary classification and does not explicitly localize flames/smoke. In real surveillance systems, temporal cues and spatio-temporal modeling can further improve robustness and reduce false alarms. Moreover, the curated dataset is assembled from multiple sources; a more systematic study of source bias and label noise, as well as domain generalization settings (without accessing target images during training), are promising directions for future work.

The performance gap between the two transfer directions suggests asymmetric domain shift. Outdoor → Indoor is more challenging, potentially due to diverse indoor lighting and background clutter. Future work may consider class-conditional alignment, stronger backbones, and more robust augmentation to further improve cross-domain robustness.

Despite the significant improvements achieved via unsupervised domain adaptation, the current framework relies on frame-level binary classification, which may ignore temporal dynamics that could further suppress intermittent false alarms. In future work, incorporating spatio-temporal modeling will be explored. Ultimately, the robust cross-domain detection capabilities demonstrated in this study establish a strong foundation for vision-based situational awareness. By reliably detecting leakage-induced fires across varying domains without site-specific re-training, this system can be seamlessly integrated into emergency response protocols for hazardous chemical transportation, thereby minimizing response latency and safeguarding human lives.

## 7. Conclusions

This paper investigates cross-domain fire and smoke detection across indoor and outdoor scenes. To address severe appearance shifts and negative transfer, a dedicated benchmark is curated for cross-domain evaluation, and several representative domain-adaptation objectives are systematically examined in this setting. The experimental results show that explicit domain alignment is beneficial for cross-domain fire detection: DANN provides the strongest target-domain accuracy in both transfer directions, while MK-MMD achieves the best fire-class F1-score on Outdoor → Indoor. The study also reveals a clear asymmetric transfer difficulty between the two directions, indicating that cross-domain fire detection should be evaluated beyond a single source-target setting. Since the benchmark is organized from publicly available datasets, the curated split definitions and implementation code will be released to support reproducible future comparisons. Future work will explore source-free domain adaptation for restricted deployments, and further investigate real-world constraints such as inference latency, target-free generalization, and stricter false-alarm control in safety-critical systems.

## Figures and Tables

**Table 1:** Dataset statistics (image counts) by domain and class.

Domain	Fire	No-Fire	Total
Indoor	40,124	19,033	59,157
Outdoor	24,489	40,050	64,539
All	64,613	59,083	123,696

**Table 2:** Train/test split statistics (image counts) by domain and class.

Domain	Split	Fire	No-Fire	Total
Indoor	Train	32,099	15,224	47,323
Indoor	Test	8025	3809	11,834
Outdoor	Train	19,591	31,890	51,481
Outdoor	Test	4898	8160	13,058
All		64,613	59,083	123,696

**Table 3:** Summary of the main hyperparameter configurations used in the experiments.

Parameter	Value/Setting
CNN Backbone	ResNet-18 (ImageNet Pretrained)
Optimizer	AdamW
Learning Rate	$5\times 10^{-5}$
Weight Decay	$10^{-4}$
Batch Size	32
Training Epochs	50
Learning Rate Schedule	Cosine Annealing
Discriminator Hidden Dimension	256
Discriminator Dropout Rate	0.2
MK-MMD Kernel Bandwidths	{1, 2, 4, 8, 16}
GRL Maximum Coefficient ($\lambda_{max}$)	1.0
MK-MMD Trade-off Weight ($\lambda_{mmd}$)	0.1
IM Trade-off Weight ($\lambda_{im}$)	0.1

**Table 4:** Small-scale parameter sensitivity analysis for selecting the optimal adaptation weights $\lambda_{mmd}$ and $\lambda_{im}$ on the Indoor → Outdoor task. The configuration in bold denotes the final selection used in all experiments.

$\lambda_{\mathrm{mmd}}$	$\lambda_{\mathrm{im}}$	Accuracy (%)	F1-Score (%)
0.01	0.1	86.84	86.45
0.5	0.1	86.90	85.55
0.1	0.01	87.25	86.91
0.1	0.5	85.45	84.80
**0.1**	**0.1**	**88.53**	**87.79**
1.0	1.0	83.15	82.20

**Table 5:** Main results on cross-domain fire detection. The best accuracy (Acc, %) and F1-score for the fire class (F1, %) are reported on the target test set.

Method	Indoor → Outdoor		Outdoor → Indoor	
	Acc (%)	F1 (%)	Acc (%)	F1 (%)
DANN	89.44	89.06	79.10	77.50
MK-MMD	86.54	86.05	79.10	78.04
MCD	81.49	80.78	73.93	72.49
DANN + MK-MMD + IM	88.53	87.79	–	–
MMD	71.30	–	70.47	–

**Table 6:** Best checkpoint summary for each run. Acc/F1/P/R are reported in % on the target test set when available. For MMD logs, Acc corresponds to val_acc.

Direction	Method	Epoch	Acc	F1	P/R
I → O	DANN	30	89.44	89.06	91.46/86.78
I → O	MK-MMD	50	86.54	86.05	88.40/83.83
I → O	MCD	5	81.49	80.78	83.17/78.53
I → O	DANN + MK-MMD + IM	41	88.53	87.79	92.87/83.24
I → O	MMD	16	71.30	–	–
O → I	DANN	27	79.10	77.50	86.41/70.25
O → I	MK-MMD	49	79.10	78.04	84.50/72.50
O → I	MCD	13	73.93	72.49	78.87/67.07
O → I	MMD	7	70.47	–	–

## Data Availability

The data presented in this study were derived from the following resources available in the public domain: https://www.kaggle.com/datasets/amerzishminha/forest-fire-smoke-and-non-fire-image-dataset?resource=download-directory (accessed on 4 February 2026); https://www.kaggle.com/datasets/mohnishsaiprasad/forest-fire-images (accessed on 4 February 2026); https://web.mit.edu/torralba/www/indoor.html?utm_source=chatgpt.com (accessed on 4 February 2026); https://www.kaggle.com/datasets/pengbo00/home-fire-dataset? (accessed on 4 February 2026); https://zenodo.org/records/15826133 (accessed on 4 February 2026); https://universe.roboflow.com/sih-hisav/indoor-fire-detection (accessed on 4 February 2026); https://universe.roboflow.com/object-detection-7qn6l/fire-smoke-indoor (accessed on 4 February 2026); https://universe.roboflow.com/ziad-f3wym/fire-detection-0anpm (accessed on 4 February 2026).

## Glossary

**Notation used in this paper.**

**Symbol**	**Description**
G(·)	feature extractor (CNN backbone)
C(·)	label classifier head
D(·)	domain discriminator
f=G(x)	feature vector of an input image
p(y|x)	predicted class distribution (softmax)
d∈{0,1}	domain label (source/target)
$L_{cls}$	source classification loss
$L_{dom}$	domain-adversarial loss (DANN)
$L_{mmd}$	feature alignment loss (MK-MMD)
$L_{im}$	information maximization loss
